# A Bioinformatics Resource for TWEAK-Fn14 Signaling Pathway

**DOI:** 10.1155/2012/376470

**Published:** 2012-05-09

**Authors:** Mitali Bhattacharjee, Rajesh Raju, Aneesha Radhakrishnan, Vishalakshi Nanjappa, Babylakshmi Muthusamy, Kamlendra Singh, Dheebika Kuppusamy, Bhavya Teja Lingala, Archana Pan, Premendu Prakash Mathur, H. C. Harsha, T. S. Keshava Prasad, Gerald J. Atkins, Akhilesh Pandey, Aditi Chatterjee

**Affiliations:** ^1^Institute of Bioinformatics, International Tech Park, Bangalore 560066, India; ^2^Amrita School of Biotechnology, Amrita University, Kollam 690525, India; ^3^Department of Biotechnology, Kuvempu University, Shankaraghatta 577451, India; ^4^Department of Biochemistry and Molecular Biology, Pondicherry University, Puducherry 605014, India; ^5^Centre of Excellence in Bioinformatics, School of Life Sciences, Pondicherry University, Puducherry 605014, India; ^6^Bone Cell Biology Group, Discipline of Orthopaedics and Trauma, University of Adelaide and The Hanson Institute, Adelaide, 5002 SA, Australia; ^7^McKusick-Nathans Institute of Genetic Medicine, Johns Hopkins University School of Medicine, Baltimore, MD 21205, USA; ^8^Department of Biological Chemistry, Johns Hopkins University School of Medicine, Baltimore, MD 21205, USA; ^9^Department of Oncology, Johns Hopkins University School of Medicine, Baltimore, MD 21205, USA; ^10^Department of Pathology, Johns Hopkins University School of Medicine, Baltimore, MD 21205, USA

## Abstract

TNF-related weak inducer of apoptosis (TWEAK) is a new member of the TNF superfamily. It signals through TNFRSF12A, commonly known as Fn14. The TWEAK-Fn14 interaction regulates cellular activities including proliferation, migration, differentiation, apoptosis, angiogenesis, tissue remodeling and inflammation. Although TWEAK has been reported to be associated with autoimmune diseases, cancers, stroke, and kidney-related disorders, the downstream molecular events of TWEAK-Fn14 signaling are yet not available in any signaling pathway repository. In this paper, we manually compiled from the literature, in particular those reported in human systems, the downstream reactions stimulated by TWEAK-Fn14 interactions. Our manual amassment of the TWEAK-Fn14 pathway has resulted in cataloging of 46 proteins involved in various biochemical reactions and TWEAK-Fn14 induced expression of 28 genes. We have enabled the availability of data in various standard exchange formats from NetPath, a repository for signaling pathways. We believe that this composite molecular interaction pathway will enable identification of new signaling components in TWEAK signaling pathway. This in turn may lead to the identification of potential therapeutic targets in TWEAK-associated disorders.

## 1. Introduction

TWEAK (*TNFSF12*) is a cell surface-associated type II transmembrane protein (249 amino acids) belonging to the Tumor Necrosis Factor (TNF) superfamily [1]. Transmembrane TWEAK is processed into a secreted 156-amino-acid form, which adopts a homotrimeric conformation. The human TWEAK gene is located at chromosome 17p13.1 [1]. TWEAK mRNA has been reported to be expressed in several tissue types, such as heart [2], brain [3, 4], kidney [5, 6], and also in mononuclear blood cells [7]. Its protein product has multiple biological activities, including stimulation of cell growth and angiogenesis [8], induction of inflammatory cytokines [9, 10] and stimulation of apoptosis [11, 12]. It has been shown to be involved in the induction of cellular proliferation in liver cells [13], osteoblasts [14], astrocytes [15], synoviocytes [16], kidney cells [17, 18], and skeletal muscle [19]. TWEAK may also play a role in the cellular differentiation of osteoclasts; however it remains controversial whether this effect is direct [20] or indirect, via effects on the osteoblastic stromal cell expression of RANKL (*TNFSF11*) [21]. TWEAK also plays a role in inducing glioma cell survival via imparting resistance to cytotoxic agents [3, 22]. TWEAK serves a dual role in angiogenic regulation. It induces the endothelial cell survival and can be a potential proangiogenic or antiangiogenic agent based upon the presence of angiogenic promoting cytokines [8, 23]. Additionally, an apoptotic effect of TWEAK has been observed in endometrial cancers [24] and peripheral blood monocytes [25, 26]. The apoptotic function of TWEAK appears to be mediated via the induced secretion of TNF*α*, with the TNF*α*-TNF*α* receptor complex, thereafter inducing autocrine cellular apoptosis by activating the RIPK1-FADD-Caspase-8 complex [11, 27]. TWEAK was first described as an apoptotic factor by interacting with DR3 (*TNFRSF25*). However, there were conflicting reports to the TWEAK-DR3 interaction [28, 29]. Hence, we chose to exclude TWEAK-DR3 pathway analysis from our study. In addition, TWEAK has been reported to interact with CD163 [30]; however, the downstream effect of this interaction remains to be explored.

TNFRSF12A (tumor necrosis factor receptor super-family, member 12A), also known as FGF-inducible 14 (Fibroblast Growth Factor-Inducible-14/Fn14), has been established to date to be the major, if not sole, receptor for TWEAK [12, 31, 32]. Fn14 is the smallest member of the TNFR superfamily described so far, and it appears to signal via recruitment of several different TNFR-associated factors [33]. This molecule has been reported to be expressed in variety of organs including the heart [34, 35], kidney [6, 36], and lung [37]. The cytoplasmic domain of Fn14, like other members of the TNFR superfamily, does not contain consensus amino acid sequences characteristic of domains with enzymatic activity. TWEAK binds with high affinity to Fn14 [12, 31]. This interaction can stimulate a variety of biological responses, depending on the cell type analyzed. Winkles et al. (2008) hypothesized two modes of TWEAK-Fn14 (ligand-receptor) interaction: (i) the ligand-dependent interaction, which involves the higher concentration of homotrimeric TWEAK, that binds to low concentration of Fn14 in a heterohexameric complex [38, 39], and (ii) ligand-independent interaction when the ligand concentration is lower than the receptor concentration. Here, the free receptors homotrimerize to activate the downstream events [38]. Three notable signaling cascades have been reported under TWEAK-Fn14 interactions. They are the canonical and noncanonical NF-*κ*B pathways [21, 33, 34, 40] and the MAPK pathway [41–43] with possible binding to TRAF proteins.

The differential effects of TWEAK on disease pathogenesis have been proposed by various groups. These diseases include autoimmune disorders [16, 21, 44, 45], neurological disorders [46, 47], periodontal disease [7], and cancers [3, 22, 24, 48–50]. Because of its multifunctional properties, TWEAK is also being considered for use in therapeutics [51]. It is also being considered as a potential early and prognostic biomarker for conditions such as kidney injury [52, 53], SLE [54], atherosclerosis [55, 56], cardiovascular disorders [57–59], immune preconception marker [60], and abdominal aortic aneurysms [61]. Although the results obtained to date are captivating, it is clear that additional studies are required to determine whether TWEAK, and/or Fn14 could be novel molecular targets for developing anticancer and antiautoimmune therapeutic agents in humans.

Thus, given its importance in the field of biomedical research, we carried out an extensive and iterative compilation of TWEAK-Fn14 signaling pathway by literature mining. Information gathered on protein-protein interactions, posttranslational modifications, protein transportation events, and regulation of gene expression, which are stimulated by TWEAK were compiled into a signaling pathway using a visualization tool, PathVisio [62]. Our compiled data will be useful for the scientific community to explore, further, the role of TWEAK in differential disease pathogenesis, in biomarker development. Using similar approach, we have also developed signaling pathways on leptin [63] receptor activator for nuclear factor  *κ*B ligand (RANKL) [64] and follicle stimulating hormone (FSH) [65]. In the current study, we have generated a reaction map of TWEAK signaling pathway, which is available for visualization at NetSlim [66] (http://www.netpath.org/netslim/), an accessory resource for visualization of NetPath pathways [67].

## 2. Methods

**Figure 1:**
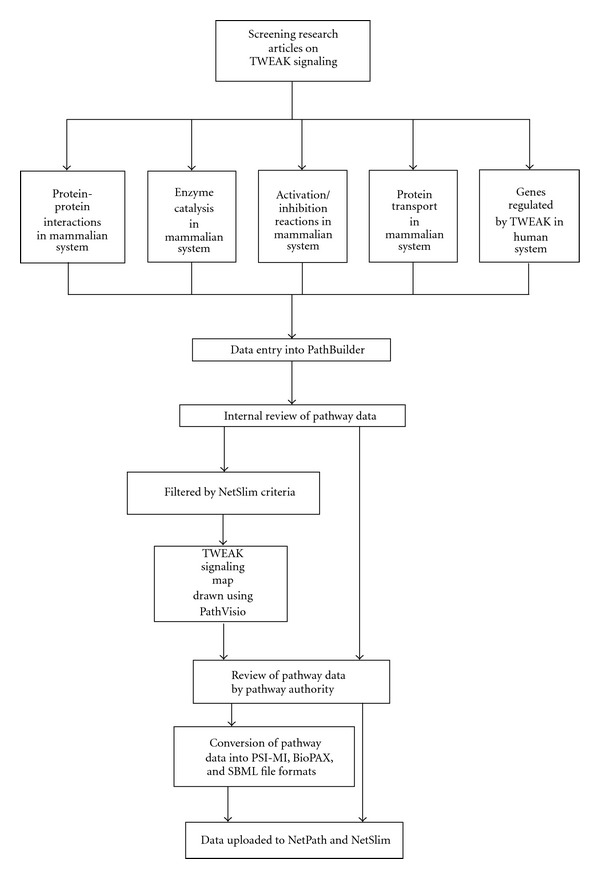
Workflow of the study. Articles were screened based on TWEAK stimulus and the molecular events were added to PathBuilder. Data were then transferred to NetPath repository. With the help of PathVisio tool, the reactions were used to generate the TWEAK signaling map (http://www.netpath.org/netslim/tweak_pathway.html).

PubMed searches were performed using TWEAK or Fn14 and their alternate names as keywords to retrieve relevant articles pertaining to TWEAK signaling. The articles were screened to capture molecular reactions stimulated by TWEAK in mammalian cells as compared to the corresponding unstimulated state. Thereafter, with the use of an in-house developed software, PathBuilder [68] that enables conversion of pathway data into standard community formats, namely, PSI-MI, BioPAX, and SBML formats, we annotated biological information and reactions pertaining to TWEAK signaling. These included protein-protein interactions, enzyme-substrate reactions, gene regulation events, and also various activation/inhibition reactions under a TWEAK stimulus. These data after manual revision were exported to the NetPath database, (http://www.netpath.org/), a manually assembled resource for signaling pathways generated by our group [67] which provides the criteria for data compilation. The entire workflow is briefly summarized in Figure 1.

### 2.1. Protein-Protein Interactions

The protein-protein interactions gathered from several experimental platforms were cataloged from literature into either binary or complex interactions. A binary interaction represents the interaction of two proteins either in homomeric or heteromeric form. A complex protein interaction comprises reactions involving more than two proteins, which again can be either homomeric or in heteromeric. For every protein-protein interaction, we documented information on subcellular localization, the experimental method used, the name and species of cell models, and finally, the hyperlinked PubMed identifier for the corresponding publication.

### 2.2. Catalytic Reactions

We compiled the posttranslational modifications under TWEAK stimulus and mapped them to their corresponding protein sequences in the RefSeq database. Further, activation or inhibition of the substrate in response to the stimulus was also compiled. The notable modifications chosen were phosphorylation, acetylation, ubiquitination, sumoylation, protein degradation, and methylation. The mode of amassment was of two types, direct and indirect. Direct included those reactions where the enzyme has been reported for the specific type of protein (substrate) modification. Indirect reactions include those where the type of modification is experimentally proved; however no information exists about its immediate upstream enzyme. The features added for the enzyme-substrate reactions include the type of posttranslational modification, the site and residue of each modification, the source of protein, the species used, and cellular localizations of the respective reaction. Additionally, we have incorporated a PubMed identifier as a hyperlink pertaining to the reaction.

### 2.3. Activation-Inhibition Reactions

Several molecules, including the Caspases-3, -7, and -8 (*CASP3*, *CASP7* and *CASP8*) [24], JUN [20, 29, 69, 70], and NIK (*MAP3K14*) [52, 71], were activated, whereas STAT1 was inhibited under TWEAK stimulus [72]. These molecules do not abide by the enzyme-substrate reactions and protein-protein interaction parameters as described previously and thus cannot be connected directly to the main frame of the TWEAK pathway and are referred to as orphan molecules. We have provided the source of protein, subcellular localization, species, and cell line in which the activation or inhibition event was reported. The PubMed identifier hyperlinked for every event was also provided. 

### 2.4. Protein Translocation Events

Subcellular transportation events of proteins under the influence of TWEAK reported to date, with appropriate Gene Ontology terms, were added into the PathBuilder tool. These events were selected on the basis of the posttranslational modifications, physical interaction or regulatory events. A TWEAK stimulus resulting in subcellular relocalization of proteins was evidenced by fluorescent microscopy and immunohistochemical studies. In addition to a particular protein's altered localization, we have also documented the source of protein and cell lines used. The criteria followed were same as mentioned in the earlier section.

### 2.5. Gene Expression Data

We have documented genes whose expressions are regulated by the TWEAK-Fn14 signaling in humans. Such genes that have been identified by various groups at the mRNA level were catalogued from DNA microarray and nonarray-based experiments such as Northern blotting, quantitative RT-PCR, or SAGE.

Further, we have included transcription regulators (transcription factors, or their coactivators/corepressors) downstream of TWEAK-Fn14 stimulus. Some of these transcription regulators are involved in the regulation of the genes (mentioned above) upon TWEAK signaling. This too has been documented and depicted in the pathway diagram. Such transcriptional regulators have been identified by approaches such as chromatin immunoprecipitation assays, electrophoretic mobility shift assays, gene silencing, and promoter activity assays in TWEAK-Fn14 signaling.

### 2.6. Selection of Sample and Species Types

Data for protein-protein interactions, catalytic reactions, and transportation events were collected from diseased or normal mammalian sources that include humans and their orthologs. However, for the gene regulatory reactions, we considered normal human cells only.

### 2.7. Generation of the TWEAK-Fn14 Pathway Map

The manually assembled data in PathBuilder were compiled and imported into NetPath (explained under methodology) [67]. A composite map of pathway reactions pertaining to TWEAK signaling were generated using PathVisio [62] by following the NetSlim parameters as have been employed earlier by our group [66]. NetSlim (http://www.netpath.org/netslim/) is a tributary of NetPath, which projects or summarizes only stringent reactions pertaining to the specific receptor-ligand complex compiled in a particular study, for example, TWEAK in this case. The criteria for selecting high confidence reactions for TWEAK pathway are provided in the NetSlim database (http://www.netpath.org/netslim/criteria.html).

## 3. Results and Discussion

We show here for the first time in any scientific repository a pathway illustration under TWEAK stimulus. Given the multifunctional properties of TWEAK, we carried out a comprehensive literature search under TWEAK stimulus followed by manual amassment, thereafter reviewing and adding the data into NetPath database [67].

### 3.1. TWEAK-Stimulated Data in NetPath

**Figure 2:**
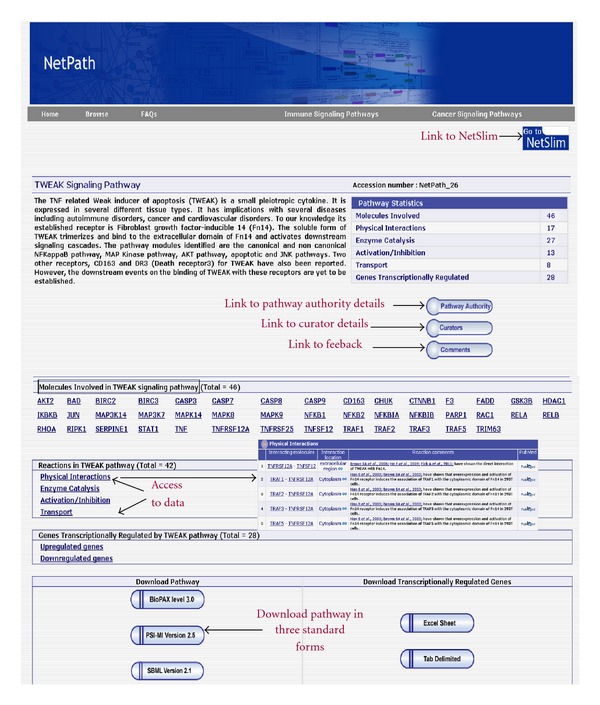
Illustration of the TWEAK page in NetPath. The image provides an outline of the TWEAK pathway as visualized in the NetPath webpage. The figure shows the statistical details of TWEAK pathway-based reactions (right upper corner). Under, “Molecules involved in TWEAK signaling pathway”, each molecule has been linked to its respective NetPath page. Tabs have been provided which leads to the details of the “pathway authority,” “curators,” and “comments” tab—where the users can provide their feedback and the reaction tabs. “Access to data” indicates links to the events regulated by TWEAK. The pathway can be downloaded from the three standard formats provided at the bottom of the page.

Fifty-eight articles were found relevant to our amassment criteria from amongst 357 articles published between 1997 and 2011. This study led to the documentation of 46 unique proteins amid which 17 were associated with protein-protein interactions, 20 involved in enzyme-substrate reactions, 13 involved in activation-inhibition reactions, and 8 were identified to be translocated from cytoplasm to nucleus. There were 28 genes identified to be differentially regulated under TWEAK stimulus in human systems. An overview of the TWEAK pathway in “NetPath” is summarized in Figure 2, which can be accessed from http://www.netpath.org/pathways?path_id=NetPath_26.

### 3.2. TWEAK-Stimulated Signaling Pathway under NetSlim

**Figure 3:**
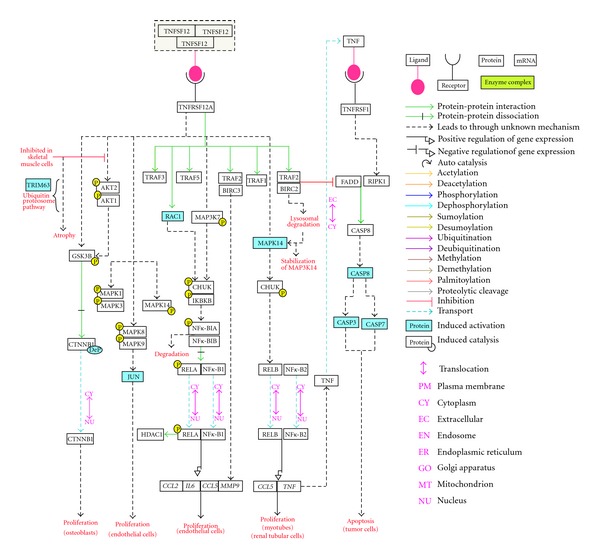
TWEAK signaling pathway: illustration of the TWEAK signaling pathway as visualized in NetSlim web page. Each molecule is linked to its corresponding page in NetPath. Each reaction is linked to its respective PubMed citation. Dashed arrows represent the downstream reactions leading to the corresponding events while the solid arrows indicate the direct association between the indicated molecules. Gene symbol(s) has been used to denote proteins in the pathway map (refer to Synonymous for common names).

The data for visualization of TWEAK signaling pathways were obtained after filtering NetPath data using NetSlim parameters. A total of 36 molecules involved in 42 reactions are visually depicted in the TWEAK pathway in NetSlim. The map generated is provided in Figure 3 and can be downloaded from http://www.netpath.org/netslim/tweak_pathway.html. The pathway illustration is also accessible at wikipathways from http://www.wikipathways.org/index.php/Pathway:WP2036. 

### 3.3. Data Availability and Reactions

The TWEAK data in NetPath are available freely and can be used by the scientific community. The data are represented in various standard exchange formats that include Biological PAthway eXchange (BioPAX) [73], Systems Biology Markup Language (SBML) [74] and Proteomics Standards Initiative Molecular Interaction (PSI-MI) [75] language formats. The PSI-MI is a community standard language for molecular interaction data used for data comparison and exchange. However, SBML is a machine readable format for representing biological models. BioPAX is another standard language that has features compatible with SBML and PSI-MI formats. The TWEAK signaling representation can be downloaded from the NetSlim database in various formats, such as “gpml”, “GenMAPP”, “png”, and “pdf”. The gene regulation data are made available in tab-limited and Microsoft Excel formats.

### 3.4. Summary of the TWEAK Pathway Reactions

A pathway module is defined as an established cascade of events that takes place inside a cell that has no defined boundaries and is part of a generic network. Some well-known modules are the NF-*κ*B, MAPK, the JNK pathways and the PI3K/AKT pathway modules. A schematic model of the TWEAK pathway with identified pathway modules is represented in Figure 3. The TWEAK-Fn14 complex binds to the TRAF molecules, TRAF 1, 2, 3, and 5. However, the downstream signaling cascade(s) that proceeds upon the association of TWEAK-Fn14 complex and TRAF 1/3/5 (*TRAF1*, *TRAF3*, *TRAF5*) is unavailable due to the lack of published studies to date. It was possible to decipher the downstream events following the formation of the TRAF2-cIAP1 (*BIRC2*) complex. This complex possibly undergoes Cathepsin B mediated degradation. The degradation of the TRAF2-cIAP1 complex leads to the stabilization of NIK and activation of the noncanonical NF-*κ*B pathway as represented in the model. The degradation of the TRAF2-cIAP1 complex also leads to the activation of the caspase pathway resulting in the apoptosis of tumor cells [11, 27]. Ikner and Ashkenazi [11] have shown that TWEAK activates apoptosis through the formation of a RIP1-FADD-caspase8 complex by TNF*α* mediated signaling, wherein cIAP1 plays a crucial role. A possible role of TWEAK has been reported in bone and cartilage damage. In fibroblast-like synoviocytes, TWEAK activates TRAF2 and cIAP2 proteins which in turn activate the MMP9 expression [76]. Experimental evidence indicates that TWEAK-Fn14 complex formation leads to the activation of p38 (*MAPK14*), ERK1/2 (*MAPK3*/*MAPK1*), JNK1/2 (*MAPK8*/*MAPK9*), and TAK1 (*MAP3K7*). No evidence has been obtained from existing literature for further direct downstream targets of p38 and ERK1/2. However, the activation of TAK1 leads further to the downstream activation of the NF-*κ*B/p65/p50 pathway. Also, RAC1 has been reported to interact directly with the TWEAK-Fn14 complex leading to activation of the NF-*κ*B pathway. Activation of AKT via phosphorylation has been observed under TWEAK stimulus with an exception in the case of skeletal muscle [41]. AKT phosphorylation leads further to the inactivation of GSK3*β* resulting in an increase in levels of phospho-GSK3*β* and active (dephosphorylated) *β*-catenin1 (*CTNNB1*). The cytoplasmic accumulation of active  *β*-catenin1 results in its nuclear translocation [14]. In addition to binding of TWEAK with Fn14, we have also documented the binding of CD163 [30] and DR3 [28] with TWEAK. Since the interaction between TWEAK and DR3 remains controversial [28, 29] and the downstream consequences of a possible TWEAK-CD163 interaction remain to be explored, the pathway illustration does not elaborate on the downstream events for these interactions.

## 4. Conclusions

The ever increasing experimental data on the various molecular events taking place following ligand-receptor interactions, in this case between TWEAK and Fn14, make it essential to have a repository for the data and also to create a signaling pathway summary. Our current work, which incorporates the TWEAK-signaling pathway data into “NetPath”, would open avenues for further studies of TWEAK-associated proteins and related disorders, such as cancers and autoimmune diseases. To our understanding, this study compiles for the first time TWEAK induced signaling events; these include (i) the inactivation of GSK3*β*  followed by dissociation of  *β*-catenin1 [14], (ii) the proapoptotic nature of TWEAK mediated through the expression of TNF*α*, which further leads to the activation of caspase8 [11], and (iii) the association between TWEAK and cIAP proteins (1 and 2) [11, 76]. We believe that our data will be informative in therapeutic studies, in selecting/pathological events and the simultaneous production of blocking agents. Importantly, the “NetPath” repository is dynamic and will allow a progressive update of relevant data, as more published literature is introduced. In addition to the direct usage of the data stored in the “NetPath” database, data can also be exported to other databases, enabling comparison and sharing amongst multiple databases, especially those which have compatible language formats, such as BioPAX [73]. Despite the minimal amount of data, ours can also be used in the overlay of various high-throughput data enabling pathway analysis and can be accessed by any pathway resource to generate a customized pathway. We are currently working on the features in “NetPath”, which are incompatible with BioPAX, especially the ontology hierarchy that has been proposed by the BioPAX group [73]. To our knowledge, our compilation of data in “NetPath” will allow, for the first time for any available scientific repository, a comprehensive study of the TWEAK pathway and its potential biomedical applications.

## Abbreviations

BioPAX: Biological PAthway eXchange
PSI-MI: Proteomics Standards Initiative for Molecular Interaction
SBML: Systems Biology Markup Language
TNF*α*: Tumor necrosis factor
IFN-*γ*: Interferon gamma
STAT-1: Signal transducer and activator of transcription 1, 91 kDa
HDAC-1: Histone deacetylase 1
GSK3*β*: Glycogen synthase kinase 3 beta
FOXO1a: Forkhead box O1
MTOR: Mechanistic target of rapamycin (serine/threonine kinase)
RAC1: Ras-related C3 botulinum toxin substrate 1
p38: Mitogen-activated protein kinase 14
AKT1: v-akt murine thymoma viral oncogene homolog 1
AKT2: v-akt murine thymoma viral oncogene homolog 2
TRAF1: TNF receptor-associated factor 1
TRAF2: TNF receptor-associated factor 2
TRAF3: TNF receptor-associated factor 3
TRAF5: TNF receptor-associated factor 5
TNF: Tumor necrosis factor
RIPK1: Receptor (TNFRSF)-interacting serine-threonine kinase 1
RELA: v-rel reticuloendotheliosis viral oncogene homolog A
RELB: v-rel reticuloendotheliosis viral oncogene homolog B
CASP3: Caspase3
CASP7: Caspase 7
CASP8: Caspase 8
IKBKB: Inhibitor of kappa light polypeptide gene enhancer in B-cells, kinase beta.
